# Supplementary material for: Role and mechanism of NCAPD3 in promoting malignant behaviors in gastric cancer
Source: Front Pharmacol. 2024 Apr 22;15:1341039. doi: 10.3389/fphar.2024.1341039 (PMC11070777; doi:10.3389/fphar.2024.1341039)
Supplement: Supplementary file 11 [file DataSheet2.ZIP › GSEA/immunologic/my_analysis.Gsea.1599462003817/GSE37416_CTRL_VS_3H_F_TULARENSIS_LVS_NEUTROPHIL_UP.html]

Details for gene set GSE37416\_CTRL\_VS\_3H\_F\_TULARENSIS\_LVS\_NEUTROPHIL\_UP[GSEA]

|  || Dataset | filtered\_dataset.sample\_info.cls#WT\_versus\_NCAPD3\_MUT |
| Phenotype | sample\_info.cls#WT\_versus\_NCAPD3\_MUT |
| Upregulated in class | WT |
| GeneSet | GSE37416\_CTRL\_VS\_3H\_F\_TULARENSIS\_LVS\_NEUTROPHIL\_UP |
| Enrichment Score (ES) | 0.41165778 |
| Normalized Enrichment Score (NES) | 1.785647 |
| Nominal p-value | 0.008264462 |
| FDR q-value | 0.9665867 |
| FWER p-Value | 1.0 |
Table: GSEA Results Summary

  

Fig 1: Enrichment plot: GSE37416\_CTRL\_VS\_3H\_F\_TULARENSIS\_LVS\_NEUTROPHIL\_UP      
 Profile of the Running ES Score & Positions of GeneSet Members on the Rank Ordered List

  

| SYMBOL | TITLE | RANK IN GENE LIST | RANK METRIC SCORE | RUNNING ES | CORE ENRICHMENT || 1 | 55727 | BTBD7 | 38 | 0.954 | 0.0552 | Yes |
| 2 | 140707 | BRI3BP | 40 | 0.947 | 0.1365 | Yes |
| 3 | 2926 | GRSF1 | 75 | 0.849 | 0.1855 | Yes |
| 4 | 55900 | ZNF302 | 145 | 0.740 | 0.2000 | Yes |
| 5 | 7733 | ZNF180 | 154 | 0.728 | 0.2573 | Yes |
| 6 | 92342 | METTL18 | 322 | 0.579 | 0.1872 | Yes |
| 7 | 84135 | UTP15 | 330 | 0.576 | 0.2321 | Yes |
| 8 | 2342 | FNTB | 344 | 0.570 | 0.2721 | Yes |
| 9 | 11019 | LIAS | 409 | 0.533 | 0.2721 | Yes |
| 10 | 801 | CALM1 | 424 | 0.518 | 0.3069 | Yes |
| 11 | 55870 | ASH1L | 436 | 0.512 | 0.3433 | Yes |
| 12 | 23383 | MAU2 | 459 | 0.500 | 0.3708 | Yes |
| 13 | 84674 | CARD6 | 463 | 0.497 | 0.4117 | Yes |
| 14 | 26100 | WIPI2 | 601 | 0.423 | 0.3497 | No |
| 15 | 29890 | RBM15B | 651 | 0.400 | 0.3490 | No |
| 16 | 28996 | HIPK2 | 687 | 0.385 | 0.3572 | No |
| 17 | 90231 | KIAA2013 | 787 | 0.330 | 0.3146 | No |
| 18 | 3146 | HMGB1 | 831 | 0.295 | 0.3092 | No |
| 19 | 2150 | F2RL1 | 887 | -0.304 | 0.2959 | No |
| 20 | 10616 | RBCK1 | 1042 | -0.438 | 0.2230 | No |
| 21 | 5565 | PRKAB2 | 1094 | -0.474 | 0.2273 | No |
Table: GSEA details [plain text format]

  

Fig 2: GSE37416\_CTRL\_VS\_3H\_F\_TULARENSIS\_LVS\_NEUTROPHIL\_UP      
 Blue-Pink O' Gram in the Space of the Analyzed GeneSet

  

Fig 3: GSE37416\_CTRL\_VS\_3H\_F\_TULARENSIS\_LVS\_NEUTROPHIL\_UP: Random ES distribution      
 Gene set null distribution of ES for **GSE37416\_CTRL\_VS\_3H\_F\_TULARENSIS\_LVS\_NEUTROPHIL\_UP**
